# A rare presentation of micro-angiopathic haemolytic anaemia in a critically ill patient: a case report

**DOI:** 10.4076/1757-1626-2-6294

**Published:** 2009-06-30

**Authors:** Mark Vivian, Chris Kirwan, Mike Grounds

**Affiliations:** General Intensive Care Unit, 1^st^ Floor St James Wing, St George's HospitalTooting, London SW19 0QTUK

## Abstract

A 36-year-old woman presents to hospital peri-arrest with hypertension, sustained loss of consciousness following a tonic clonic seizure and a micropathic haemolytic anaemia on blood film. After initial resuscitation, more specialised treatment was instigated as the diagnosis became clearer but all was not as it first seemed. This case demonstrates the importance of re-examination, especially in the critically ill, in conjunction with unusual laboratory tests in order to eventually reach a rare diagnosis of a rare presentation.

## Case presentation

A previously healthy 36-year-old Caucasian female was brought by ambulance to a South London Accident and Emergency department having collapsed at home. She was transferred to the General Intensive Care Unit soon after arrival at the hospital.

A collateral history was sought from the patient's husband. She had a 3 day history of diarrhoea and vomiting but otherwise no headache, photophobia, fevers or recent travel. There was no past medical history and he could not remember any unusual complaints or prior symptoms. He had witnessed abnormal twitching of his wife's right hand and arm, which then progressed to a tonic-clonic seizure. Paramedics were called and found the patient obtunded with a Glasgow Coma Scale (GCS) score of 3/15 (E1, M1, V1).

On arrival at the Resuscitation room, her heart rate was 130 bpm, with a blood pressure 240/120 mmHg. She had been intubated with a cuffed endotracheal tube by the attending paramedics and was mechanically ventilated with an inspired FiO_2_ of 0.4. Initial examination revealed mild jaundice but no hepatosplenomegaly or lymphadenopathy. She was bleeding from her right nostril but there was no rash or obvious bruising. Her lung fields were clear and heart sounds normal. A urine dipstick revealed the presence of blood cells (+++) and protein (+++) and an electrocardiogram showed t-wave inversion in chest leads V1-5 as the only abnormality. Chest x-ray demonstrated generalised interstitial shadowing consistent with pulmonary oedema. Arterial blood gas on admission showed pH 6.74, lactate 18 and a base deficit of 23.2. Empirical therapy for suspected meningitis was started. One gram of phenytoin was given intravenously.

The patient was transferred the General Intensive Care Unit. Once stable following fluid resuscitation she underwent a computed tomography scan of her brain. This showed no evidence of acute cerebral bleed, space occupying lesion or features of raised intracranial pressure. Concurrently her formal laboratory blood tests became available (Table 1).

**Table 1.** Admission blood test results

Haemoglobin	6.6	g/dL	(12.0-16.0)	Sodium	137	mmol/L	(135-145)
Platelets	32	10^9^/L	(150-450)	Potassium	4.3	mmol/L	(3.5-4.7)
White blood cells	24.7	10^9^/L	(4.0-11.0)	Urea	13.6	mmol/L	(2.5-8.0)
Neutrophils	15.9	10^9^/L	(1.7-8.0)	Creatinine	266	μmol/L	(60-110)
INR	1.5		(0.8-1.1)	LDH	1164	U/L	(0-175)
APTT ratio	1.68		(0.85-1.15)	Glucose	13.7	mmol/L	(3.0-6.0)
Thrombin Time	17	secs	(11-16)	Bilirubin	40	μmol/L	(0-17)
Fibrinogen	1.8	g/L	(2-4)	ALT	30	U/L	(0-40)
D-dimer	2.75	mg/L	(0-0.3)	ALP	64	U/L	(35-120)
CRP	14.8	mg/L	(0-10)	Albumin	25	g/l	(35-48)
Bicarbonate	17	mmol/L	(22-32)	Gamma GT	13	U/L	(0-38)
Phosphate	3.26	mmol/L	(0.75-1.5)	CK	274	μmol/L	(30-210)

**Figure 1.**
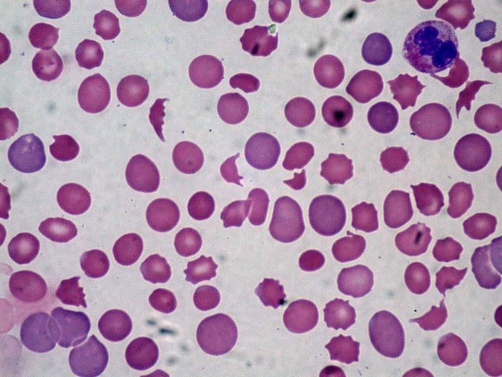
Blood film of MAHA (reproduced with permission from commons.wikimedia.org/wiki/Image: DIC_With_Microangipahic_Hemolytic_Anemia.jpg).

Lumbar puncture was not performed owing to the low platelet count. The patient's blood film confirmed a microangiopathic haemolytic anaemia (MAHA, an example of which is shown in Figure 1) with anisopoikilocytosis, marked RBC fragmentation and crenation (as well as reticulocytes, spherocytes, polychromasia and neutrophilia).

Two hours into the admission following advice from our haematology colleagues, the working diagnosis was either haemolytic-uraemic syndrome (HUS) or thrombotic thrombocytopoenic purpura (TTP). Six hours after admission the patient was administered with 500 mg methylprednisolone and underwent plasmaphoresis against Fresh Frozen Plasma.

The next morning the working diagnosis was refined as follows: verotoxin producing E.coli (VTEC) + HUS with MAHA + Hypertension induced encephalopathy and concurrent Acute Kidney Injury. The hypertension was managed with intravenous and oral anti-hypertensives. The patient received a second cycle of plasma exchange.

Repeat clinical examination demonstrated a small smooth liver edge and an abdominal ultrasound scan showed no obvious intrahepatic pathology or biliary tree and urinary system dilation or blockage. More subtle clinical findings included tight hyperpigmented skin of fingers and hands to the wrist bilaterally and a V shaped area of thickened skin to the praecordium, sclerodactyly of the hands, but not the feet, and telangiectasiae to the face and neck. There were no digital ulcers or signs of calcinosis and following review and consultation with our rheumatology and nephrology colleagues we revised the working diagnosis to scleroderma renal crisis (SRC) precipitated by diarrhoea and vomiting.

With this in mind further collateral history was sought from the husband who described his wife as stoical. However, he mentioned she had complained of ‘painful hands’ over the last two months but there was no change in finger colour. The patient's father has hyperpigmented skin to his elbows and the patient's mother suffered with diabetes mellitus.

On day four she became anuric. A chest x-ray demonstrated pulmonary oedema and bilateral pleural effusions and continuous veno-venous haemofiltration (CVVHF) was commenced. Echocardiogram demonstrated a small pericardial effusion which did not cause haemodynamic compromise.

The results of autoimmune studies, which were taken on admission prior to plasma exchange, also became available on day four and are shown in Table 2.

**Table 2.** Immunological blood test results

C3	0.5 g/L (0.75-1.65)
C4	0.08 g/L (0.14-0.54)
ADAMTS-13	20% (not pathogenic)
ANA	Positive (speckled pattern, chromosome negative)
ANCA	negative
Antiphospholipid screen	normal
IgG cardiolipin antibodies	0.8 (normal)
IgM cardiolipin antibodies	1.7 (normal)
Anti SS-A, Anti SS-B, Anti SM, Anti RNP, Anticardiolipin IgG	negative
HBV SAg, HCV antibody, CMV IgM	negative

In view of the revised diagnosis the patient was transferred to a Regional Centre with a special interest in scleroderma. Further tests and investigations were performed (Table 3). She was discharged home on day 28 but continues to require renal replacement therapy in the form of Continuous Ambulatory Peritoneal Dialysis (CAPD). Her hypertension is well controlled with Enalapril, Irbesartan and Furosemide. Six months following presentation, she is back at work on a part time basis. The final diagnosis was Diffuse Cutaneous systemic sclerosis / scleroderma with microangiopathic haemolytic anaemia and renal crisis with persistent hypertension.

**Table 3.** Results from tests performed after transfer to the specialist scleroderma centre

ANA	Positive
	>1/1000 fine speckle pattern
	>1/100 ++ nucleolar
RNA Polymerase antibody	Positive
SCL-70	Negative
Rheumatoid factor	Negative
ENA screen	Negative
Double stranded DNA antibodies	5
C3	105 mg/dL
C4	21 mg/dL
HB SAg, EBV IgG, CMV IgG, HSV1 IgG, HSV2 IgG, VZV IgG	All positive

## Discussion

Microangiopathic haemolytic anaemia (MAHA) is defined as haemolysis of erythrocytes in capillaries, leading to anaemia. It is a rare disease.

Abnormal factors (including high shear stress) lead to vascular endothelial disruption of capillaries, deposition of Fibrin mesh and platelet aggregation. This causes lysis of the red blood cells (RBCs), formation of schistocytes and RBC fragments with consumption of platelets. The resultant clinical picture is: anaemia (typically haemaglobin lower than 8 g/dL), thrombocytopoenia (platelet count < 140 × 10^9^/L) with schistocytes and helmet cells on blood film. Although not performed in our patient, a Coombs test is usually negative.

The main causes of a MAHA are: Haemolytic Uraemic Syndrome (HUS, types I - typical and II - atypical), Thrombotic Thrombocytopoenic Purpura (TTP) and Disseminated Intravascular Coagulation (DIC). There are however other causes of a MAHA which include, but are not limited to, malignant hypertension (hence the association with scleroderma renal crisis). These are summarised in Table 4 below.

**Table 4.** Summary of the causes of MAHA

Features common to all causes of MAHA	Anaemia (Hb < 8 g/dL)
	Thrombocytopoenia (platelets < 140 × 10(9)/L)
	Red Blood Cell fragments, Schistocytes and helmet cells on blood film
	Negative Coombs test (IgG autoantibodies to individual's red blood cells)
	Possible multi-system involvement
HUS type I (Shiga-like / Verotoxin associated)	Associated with Verotoxin (Shiga-like) E. coli
	O157 infection
	Prodrome of diarrhoea, often bloody, 3-5 days before onset
	Typically affects children < 5 yrs
	Commonly acute renal failure
HUS type II (Non Shiga-like)	Not thought to be associated with diarrhoea
TTP	Neurological symptoms predominant
	Acute kidney injury
	Pyrexia
Disseminated Intravas-cular Coagulation	Activation of the intravascular clotting cascade
	Consumption of clotting factors and fibrinogen
	Consumption of platelets
	Raised INR, PT, APTTR
Other causes	Aortic Stenosis / replacement valve
	Scleroderma renal crisis
	Severe glomerulonephritis malignant hypertension, pregnancy associated microangiopathy (incl. pre-eclampsia, HELLP syndrome) infective (shigella, TB, E. Coli)
	Drug related (e.g. Heparin - Heparin Induced Thrombocytopoenia (HIT))

### Initial management

Our patient was transferred rapidly to the Intensive Care Department having come through the Emergency department. She was in extremis and initial management goals were life saving resuscitation and organ support with correction of her acidosis.

We included empirical antibiotics for meningitis since differential diagnosis included sepsis with neurological involvement.

The blood results revealed thrombocytopenic anaemia with RBC fragments on blood film. The diagnosis of TTP/HUS was reached with the assistance of our haematology colleagues. Despite the abnormal clotting screen it was felt that the findings of the blood film in conjunction with the clinical picture favoured the diagnosis reached. Standard treatment for this is plasmaphoresis. If left untreated, HUS/TTP follows a progressive course leading to irreversible renal failure and death. Plasmaphoresis reverses platelet consumption responsible for thrombus formation and the sequelae that characterise this condition [1] and may be initiated even if there is uncertainty to the diagnosis [2]. As a result, the mortality rate has dropped from previous rates of 90% [3] and presently it is mostly curable.

In TTP specifically, a deficiency of, or autoantibodies to, ADAMTS-13 can lead to platelet aggregation and MAHA. In these patients, plasma exchange helps remove the acquired autoantibody, as well as large vWF multimers, helping prevent further thrombosis [3]. Steroids, such as methylprednisolone, may reduce the production of autoantibodies. Our patient received 500mg of methyl prednisolone on this basis. Normal levels of ADAMTS-13 are activities of 68-128%. However, studies show that parents of TTP patients with levels of 6-20% are usually asymptomatic and levels as low as 5-10% are still enough to prevent micro thrombi [4]. With a level of 20%, it was felt that our patient did not have TTP.

Our patient contemporaneously suffered with persistent hypertension which was initially managed with a prostacyclin infusion and oral captopril. Glomerular involvement in HUS/TTP can lead to cortical necrosis (secondary to glomerular thrombotic microangiopathy). Arterioles and interlobular arteries are affected with intimal oedema, luminal narrowing and thrombosis with necrosis of the arteriolar wall. The glomeruli appear small and shrunken, with splitting of the capillary wall and interruption of the basement membrane, which is the lesion thought to be responsible for severe hypertension [5]. Anti-hypertensive treatment was titrated to 10 ng/kg/min iloprost, 100 mg tds captopril and an infusion of labetalol. Her acute renal failure was treated with continuous veno-venous haemofiltration (CVVHF).

### ‘Secondary survey’ / review

Our initial focus was providing life-saving treatment but hyperpigmentation, sclerodactyly, thickened skin on the praecordium and telangectasiae were all noted on repeated examination once our patient was stable. Further collateral history revealed a 2-month history of painful hands and a family history of autoimmune disease.

The diagnosis of scleroderma renal crisis (SRC) becomes more likely and this was confirmed by the availability of additional investigations taken on admission over the following days.

### Scleroderma renal crisis

A comprehensive review of Scleroderma renal crisis discussed the common presenting features of SRC [6]. Table 5 conveniently summarises these features (reproduced with permission).

**Table 5.** Renal crisis classification

1. New onset hypertension (>150/85 mmHg)
2. Decrease in eGFR (>30%)
Plus any of the following corroborative features:
MAHA on blood film
New onset RBCs in urine (excluding other causes)
Flash pulmonary oedema
Retinopathy typical of acute hypertensive crisis
Oliguria or anuria
Renal biopsy showing characteristic changes (accumulation of mucin in interlobular arteries - indistinguishable from accelerated hypertension - and fibrinoid calcinosis of arterioles)

Modified slightly from original and kindly reprinted with permission [11].

As can be seen from the case history, our patient suffered from all of the features listed, with the exception of retinopathy. SRC is differentiated from TTP in systemic sclerosis (SSc) with some difficulty but can be made primarily on the presence or absence of haemorrhage, fever and hypertension [7]. A concurrent diagnosis of diffuse cutaneous systemic sclerosis (dcSSc) was also made and this was the first presentation of the disease. The diagnosis of SSc is primarily made on clinical grounds: sclerodactyly, telangectasiae, hyperpigmented skin of distal arms/hands and thickened skin to the praecordium. Additionally, the positive ANA (>1/1000 fine speckle pattern, > 1/100 ++ nucleolar) and RNA polymerase is strongly associated with the diagnosis of SRC and SSc [8]. The patient's Scl-70 was negative, which is a sensitive and highly specific marker for systemic sclerosis, yet in the cases of SRC was present in only 17% patients [6], and in this instance does not rule out SSc.

The pathogenesis of SRC is thought to be as a result of capillary endothelial activation, releasing multiple vasoactive substances, some of which are thought to be pathogenic [9]. These may work in a similar way as to that of Verotoxin in HUS. Acute loss of renal function reflects the degree of vascular damage, which is independent of the blood pressure. Acute renal tissue change was also associated with a poorer prognosis of renal function [6]. At the time of writing the results of our patients' renal biopsy are unknown.

On review of our patient, it was concluded that immunosuppressive therapy was not required immediately: SRC is a vascular pathology (as described above) that requires medical treatment in the form of ACE inhibition. This constitutes renal saving therapy and the immunosuppressive medications were stopped. In addition, careful avoidance of volume depletion (i.e. with CVVHF) was needed, due to the high peripheral vascular resistance and rapid onset of hypertension with hypovolaemia.

As a result, new treatment goals were sustained lower blood pressure, renal supportive therapy in the form of CVVHF and continued management of fluid-balance to prevent fluctuations in BP and further problems such as pulmonary oedema.

Mycophenolate Mofetil (MMF) was commenced to limit the fibrotic complications in relation to cutaneous and organ involvement [10] in dcSSc. MMF is an immunomodulating medication, specifically an inositol monophosphate dehydrogenase (IMPDH) antagonist, an enzyme essential for the de novo guanosine nucleotide synthesis. It is more commonly used to prevent transplant graft rejection and in the treatment of lupus.

We have already discussed, in retrospect, there is a strong correlation with our patient's presenting features and those documented in the literature for scleroderma renal crisis. Our patient, with anti-RNA polymerase antibody and early dcSSC was at the highest risk for developing SRC [6].

### Mortality and prognosis

There is a significant mortality associated with SRC. Survival is listed as 82% at 1 year and 59% at 5 years [6]. The index of chronic kidney damage did not correlate with prognosis in SRC, which is contrary to a wide range of renal diseases. This could be a result of the vascular pathogenesis. Patients who needed dialysis temporarily had the best prognosis and those with higher BP and younger age (as in our patient) were associated with a better renal outcome [6]. Unfortunately the overall prognosis at this time remains poor.

## Conclusion

This case is of particular interest to Intensive Care, General Medical, Renal and Haematology physicians. It is a rare presentation of a rare condition (MAHA), rendering the patient critically ill. Initially believed to be secondary to a verotoxin producing E. coli infection causing HUS/TTP, it was, on reflection of the complete clinical picture, a scleroderma renal crisis. The ultimate diagnosis was one of diffuse cutaneous systemic sclerosis (DcSSc). Our patient's management has been demonstrably correct and was altered appropriately as new evidence came to light and involved many different specialist teams. However, this case also underlines the importance of careful and repeated examination, with regular review of clinical signs, even when the patient is critically ill. This is highlighted when complex treatments such as plasmapheresis may require early transfer to another institution. These basic skills of practicing medicine will lead to the correct diagnosis even in rare cases and in the face of life threatening physiology.

## Abbreviations

bpm: beats per minute
BP: Blood pressure
CVVHF: Continuous Veno-venous Haemofiltration
DIC: Disseminated Intravascular Coagulation
HUS: Haemolytic Uraemic Syndrome
MAHA: Microangiopathic haemolytic anaemia
MMF: Mycophenolate Mofetil
RBC: Red Blood Cells
SRC: Scleroderma Renal Crisis
SSc: Systemic Sclerosis
TTP: Thrombotic Thrombocytopoenic Purpura
